# Regulation of Corneal Stromal Cell Behavior by Modulating Curvature Using a Hydraulically Controlled Organ Chip Array

**DOI:** 10.21203/rs.3.rs-3973873/v1

**Published:** 2024-02-23

**Authors:** Minju Kim, Kanghoon Choi, David Krizaj, Jungkyu Kim

**Affiliations:** 1 Department of Mechanical Engineering, University of Utah, Salt Lake City, USA; 2 Department of Ophthalmology, University of Utah School of Medicine, Salt Lake City, USA

**Keywords:** Cornea curvature, Cornea stroma chip, Hydraulically controlled curvature array chip, Curvotaxis, Cell phenotype alteration, ECM remodeling

## Abstract

Curvature is a critical factor in cornea mechanobiology, but its impact on phenotypic alterations and extracellular matrix remodeling of cornea stroma remains unclear. In this work, we investigated how curvature influences the corneal stroma using a hydraulically controlled curvature array chip. The responses of stromal cells to low, medium, and high curvatures were observed by preparing three phenotypes of corneal stromal cells: corneal keratocytes, fibroblasts, and myofibroblasts. Keratocytes exhibited phenotypic alterations in response to curvature changes, notably including a decrease in ALDH3 expression and an increase in α-SMA expression. For focal adhesion, corneal fibroblast and myofibroblasts showed enhanced vinculin localization in response to curvature, while corneal keratocytes presented reduced vinculin expression. For cell alignment and ECM expression, most stromal cells under all curvatures showed a radially organized f-actin and collagen fibrils. Interestingly, for corneal fibroblast under medium curvature, we observed orthogonal cell alignment, which is linked to the unique hoop and meridional stress profiles of the curved surface. Furthermore, lumican expression was upregulated in corneal keratocytes, and keratocan expression was increased in corneal fibroblasts and myofibroblasts due to curvature. These results demonstrate that curvature influences both the phenotype of corneal stromal cells and the structural organization of corneal stroma tissue without any external stimuli. This curvature-dependent behavior of corneal stromal cells presents potential opportunities for creating therapeutic strategies for corneal shape dysfunctions.

## 1. INTRODUCTION

Approximately 1 in 2,000 individuals experience corneal dystrophy associated with shape abnormality^1, 2^. Keratoconus is a known corneal ectasia that eventually develops into a distinctive bulge resembling a cone shape due to corneal thinning^3, 4^. Similar to keratoconus, abnormal cornea shape can be found in the plana and keratoglobus^5^. Assessment of these corneal shape abnormalities depends mainly on the corneal curvature, which is expressed in diopters (D). Typically, a healthy cornea exhibits a curvature corresponding to a radius of 7.8 mm, which is equivalent to 43 D^6^. However, in the case of Plana, the curvature falls below 36 D, signifying flattening of the corneal surface^7^. In contrast, both keratoconus and keratoglobus exhibit considerable curvatures above 45 D, indicating excessive bulging^8^. Abnormal curvature has a notable impact on the structure of the cornea, leading to alterations in extracellular matrix (ECM) arrangement. *In vivo*, the corneal stroma is orthogonally organized by 200–300 collagen lamellae surrounded by small leucine-rich proteoglycans (SLRPs). The SLRPs consist of keratin sulfate, chondroitin sulfate, and dermatan sulfate^9, 10^ and regulate both collagen organization and fibrillogenesis^11^. The structural integrity and appropriate arrangement of collagen fibers in the cornea are maintained by the interaction of SLRPs with collagen^12^. Abnormalities or deficiencies in SLRPs link to alterations in the corneal curvature, specifically keratoconus^13, 14^. The progression of keratoconus is primarily driven by disruptions in the stromal collagen structure, often leading to an excessive fibrotic response^15^. This phenomenon results in corneal haziness and cloudiness, which are characteristic features of vision loss incurred by corneal damage. Therefore, the regulation of ECM deposition and degradation is crucial for the remodeling of the corneal stroma.

In addition, the alteration in ECM regulation is closely linked to the process of cellular trans-differentiation, leading to fibrosis in the corneal stroma. During the remodeling or fibrosis process, basic fibroblast growth factor (bFGF) and transforming growth factor beta (TGF-β) play critical roles as signaling messengers that modulate cell migration, phenotypic behavior, and ECM secretion of cornea keratocytes. Specifically, TGF-β triggers the transformation of quiescent cornea keratocytes into myofibroblasts, which are contractile and actively participate in tissue repair processes, and b-FGF prompts keratocytes to differentiate into fibroblasts^16, 17^. These factors transform corneal keratocytes into fibroblasts for tissue repair. Although these biochemical pathways are well known for cell phenotypic alterations, the mechanisms underlying the development of stromal fibrosis associated with corneal shape remain largely unknown. Thus, further studies are required to understand the complex mechanisms underlying the transition of corneal keratocytes into myofibroblasts in response to diverse curvatures resembling corneal ectasia.

Curvature is a crucial factor in regulating morphological changes, guiding curvotactic cell migration, influencing cell differentiation, and orchestrating the reorganization of the actin cytoskeleton. Specifically, a curvotaxis study revealed that adherent cells on curved surfaces prefer migration and positioning within concave valleys triggered by curvature-induced traction forces^18, 19^. Further studies have shown that closely linked milli-scale curvatures are related to the self-organization of structures that emerge from collective cell migration, as evidenced by the appearance of lamellipodial and filopodial protrusions^20^. In particular, certain aspects of collective cell migration have been emphasized, including 1) cell-cell communication, 2) interactions at the cell-substrate interface, and 3) the influence of leader and follower cells in generating protrusion and traction forces^21, 22^. During collective cell migration, cells sense and react to millimeter-scale curvatures, reaching dimensions up to the millimeter scale because of the crucial aspects involved in collective migration^20–22^. These studies provide insight into how curvature affects cell migration, focal adhesion, and the reorganization of fibroblasts. However, a comprehensive analysis of the phenotypic changes and unique ECM organization of the corneal stromal cells associated with different curvatures has not yet been performed.

In this study, we utilized a microfabricated curvature array chip to investigate the impact of curvature on corneal stromal keratocytes, fibroblasts, and myofibroblasts. The stresses generated by these curvatures significantly affect quiescent keratocytes, as well as activated fibroblasts and myofibroblasts, influencing their ability to self-organize the ECM structure and cell orientation. By employing a hydraulically actuated curvature array chip, we modulated curvature levels to simulate plana, keratoconus, and keratoglobus. The effect of curvature on corneal stromal cells was assessed by examining cell proliferation, phenotypic alterations, ECM expression, focal adhesion, and mechanobiological factors using various microscopy techniques, immunostaining, and gene expression. We found that curvature profoundly alters the expression of phenotypic markers such as α-SMA and ALDH3, as well as the expression of structural extracellular matrix (ECM) components and focal adhesion molecules, indicating that a direct influence of curvature on both the phenotype of corneal stromal cells and the structural organization of the corneal stroma tissue.

## 2. RESULTS

### 2.1. Design and characterization of a curvature array chip

**Figure 1.**
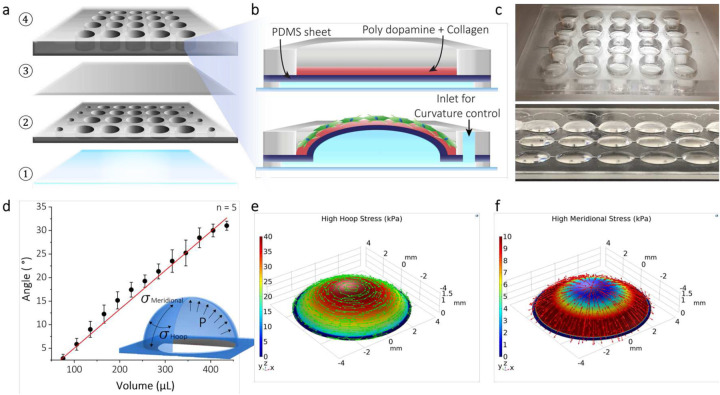
Schematic diagram of a microfabricated curvature array chip and cell seeding on the top of the curvature array chip. (a, b) Detailed curvature array chip, consisting of a medium reservoir, thin PDMS, a hydraulic control layer, and a glass substrate from 1 to 4. By injecting water into inlets using a syringe, the desired curvature is formed by inflating a thin PDMS membrane. (c) Various curvatures by controlling the injection volume. (d) Relationship between volume and angle profiles during fluid injection into the hydraulic layer. (e-f) Simulation results to present hoop and meridional stress profiles on high curvatures.

Fig. 1 illustrates the simple microfabrication process of the curvature array chip, along with its mechanical characterization. The curvature array chip consists of glass, the microfluidic channels for hydraulic actuation, a 100-μm thin polydimethylsiloxane (PDMS) sheet, and a medium reservoir as depicted in Fig. 1a and b. To maintain three distinct curvatures (low, medium, and high) steadily, the filling volume of DIW in hydraulic channels was precisely characterized to obtain proper curvatures. Fig. 1c **and Supplementary Fig. 1** show the side-view time-lapse images of the curvature. By analyzing these images, we determined the precise volume of DIW required to achieve the designated curvature as shown in Fig. 1d. Starting from the flat (control) by filling 45 μL in the hydraulic channel, 11.79° (56.25D) for high curvature, 15° (42.18D) for medium curvature, and 20.91° (33.75D) for low curvature were obtained by filling 286 μL, 212 μL, and 172 μL, respectively. In Fig. 1e and f, the curvatures generated hoop and meridional stresses to the surface. In the center area, the hoop stress was more noticeable than in other areas. However, the meridional stress was increasingly stronger when it approached the edge area. With these different properties by three selected curvatures, further investigations were performed to investigate the effects of curvature on corneal stromal cells.

### 2.2. Cell proliferation rate on the curvature array chip

**Figure 2.**
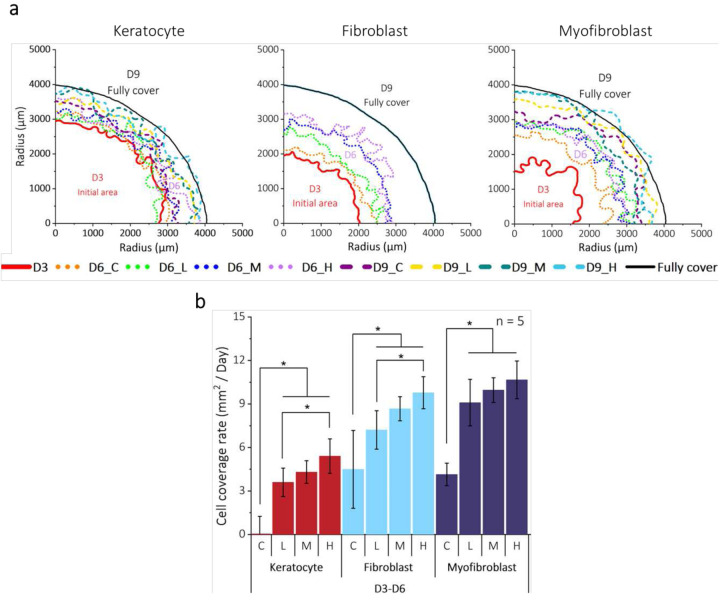
Effect of curvature on the cell coverage rate of cornea stromal cells. (a) Cell coverage contours of three corneal stroma cells on flat, low, medium, and high curvature. All cells achieved almost full coverage within six days from the starting point (D3) (b) The cell coverage rates of cells on different curvatures were assessed between D3 and D6. All three cell types exhibited increased cell coverage rates on curvature. Specifically, fibroblasts and myofibroblasts displayed a faster cell coverage rate than keratocytes.

To enhance ECM adhesion onto the PDMS surface and ensure a robust collagen coating, we treated the PDMS surface with various concentrations of polydopamine (PDA). Our titration experiments led us to determine that a 0.01% w/v PDA concentration is optimal for collagen coating following oxygen plasma treatment, as described in **Supplementary section 1 and Supplementary Fig. 2**. On a PDA-assisted collagen-coated curvature array chip, corneal stromal cells were seeded at the center of each well using a drop of cells to study the cell proliferation from top to bottom. Fig. 2a showed the contours of cell coverage of each cell type from day 3 (D3) into day 9 (D9). At D3, cornea keratocytes allowed more cell coverage than cornea fibroblasts and myofibroblasts due to the difference in the initial cell seeding density. Despite the initial coverage differences, all cell types entirely covered the 8 mm diameter circular area at D9, indicating that the activated formed cells have a higher proliferation rate. Fig. 2b shows the cell coverage rate of three distinct curvatures between D3 and D6. The coverage rate of cornea keratocytes and fibroblasts was significantly higher on curvature compared to flat (P < 0.05). Moreover, high curvature exhibited a clear difference in the coverage rate compared to low curvature. For myofibroblasts, the difference in the coverage rate between flat and low curvature was higher than that of fibroblasts. However, there was no statistically significant difference observed in the overall coverage rate on curvature between fibroblasts and myofibroblasts.

### 2.3. Effect of curvature on the cell phenotype

**Figure 3.**
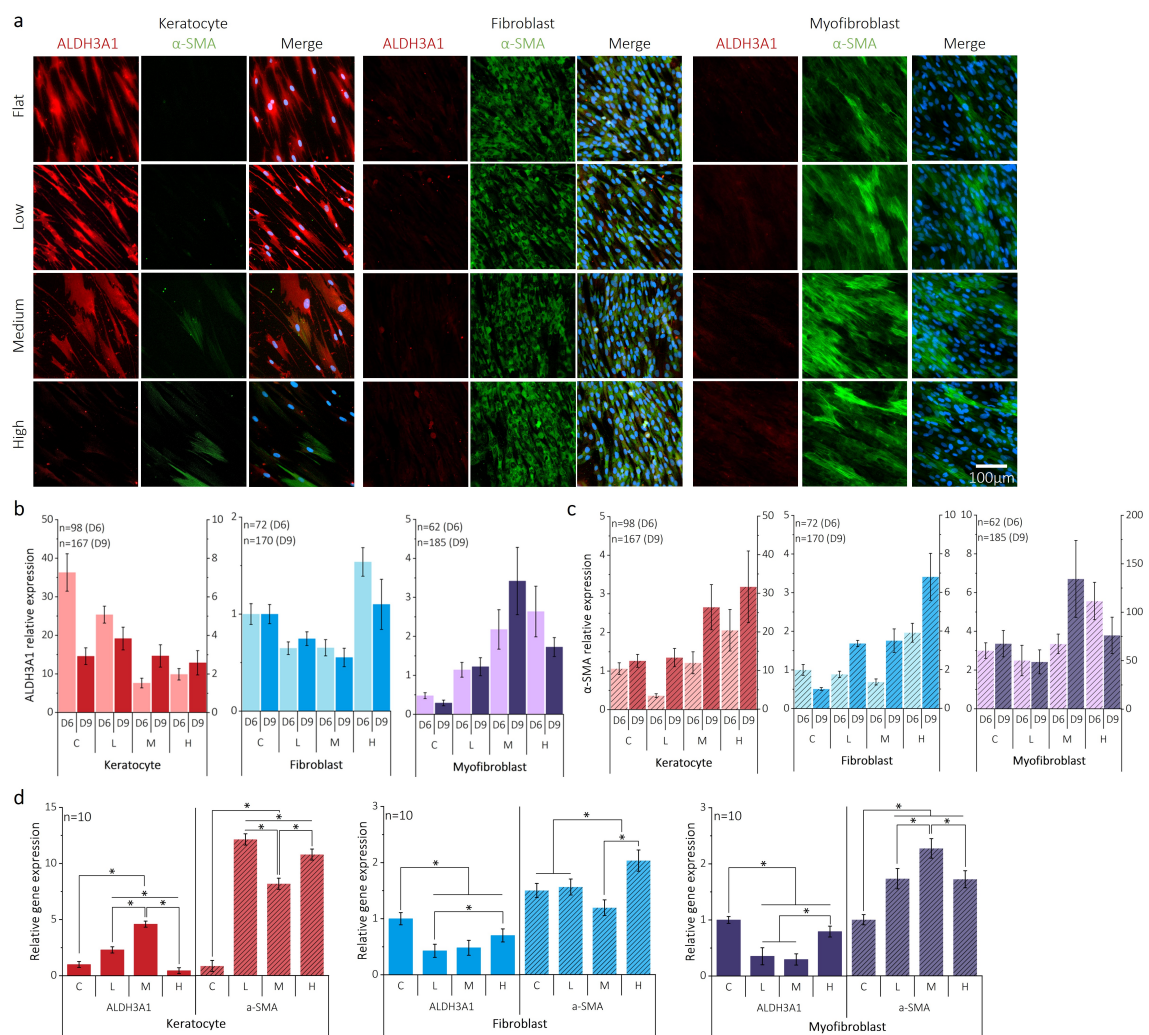
Effect of curvature on the phenotype of cornea stromal cells. (a) Immunofluorescence staining images of all cell types show the transforming cell phenotypes on curvature compared to flat using ALDH3A1 and α-SMA on Day 6 (3 days after curvature). (b-c) Quantitative mean intensity of ALDH3A1 and α-SMA on D6 and D9. ALDH3A1 expression in myofibroblasts gradually increases compared to flat and the expression level of α-SMA on D9 was consistently higher on curvature for all cell types. (d) The expression levels of cell phenotype marker genes (ALDH3A1 and α-SMA) were analyzed with different curvatures and cell types using real-time quantitative reverse transcription polymerase chain reaction on Day 9 (6 days after applying the curvature) and all samples were quantified using the 2-ΔΔCt method compared to the flat.

Stromal cells undergo essential transformations in response to corneal diseases associated with shape deformation, and phenotypic changes and stromal fibrosis can lead to various corneal disorders^23^. We confirmed the characterizations of three specific cell types for transformations of cornea stromal cells in **Supplementary section 2** and **Supplementary Fig. 3**, before applying curvature on D3. The timeline is specified in **Supplementary Fig. 4**. We observed the influence of curvature on each phenotype of corneal stromal cells by evaluating the expression of ALDH3A1 (corneal crystallin for corneal keratocyte marker) and α-SMA (fibrotic marker) on D6 and D9. For cornea keratocytes cultured without a curve, ALDH3A1 and α-SMA expression profiles were consistent with those observed in a standard culture dish. However, when cultured with a curve on D6, ALDH3A1 expression decreased and the α-SMA expression increased in response to the curvature, as presented in Fig. 3a. **Supplementary Fig. 5** shows that corneal keratocytes on D9 maintain ALDH3A1 expression at high curvature. Keratocytes showed the highest level of ALDH3A1 expression on D6 and α-SMA was highest on D9 (Fig. 3b and 3c).

Corneal fibroblasts showed low ALDH3A1 and a slightly higher α-SMA expressions in response to curvature compared to flat surface on D6 shown in Fig. 3a. However, the expression pattern of ALDH3A1 on D9, as shown in **Supplementary Fig. 5**, was significantly higher than what was observed on D6. Corneal fibroblasts showed lower expression of ALDH3A1 on both D6 and D9 when compared to flat, except at high curvature, as shown in Fig. 3b. α-SMA expression in cornea fibroblasts on D6 indicated that the only high curvature affects the cell phenotype, similar to the ALDH3A1 expression trend observed on D6, as presented in Fig. 3c. However, on D9, all curvatures of corneal fibroblasts showed significantly higher levels of α-SMA expression.

Unlike corneal keratocytes and fibroblasts, corneal myofibroblasts showed a curvature-dependent increase in expression of ALDH3A1 (Fig. 3a and 3b). Myofibroblasts on a flat surface exhibit significantly lower expression levels compared to those in keratocytes and fibroblasts on flat surfaces. In contrast to ALDH3A1 expression, the overall α-SMA expression of cornea myofibroblasts on D6 and D9 in Fig. 3c showed a significant increase in response to curvature. High curvature on D6 and medium curvature on D9 showed relatively high levels of α-SMA expression compared to other curvature and the flat surfaces. Myofibroblast α-SMA expression was initially strong, as shown in **Supplementary Fig. 3**. This result suggests that the alteration in phenotype due to curvature was relatively mild, given that myofibroblasts initially exhibited higher α-SMA expression than other cell types.

Quantitative analysis using qRT-PCR was performed to confirm expression trends of ALDH3A1 and α-SMA as shown in Figure 3d. ALDH3A1 expression levels in corneal keratocytes was higher under low and medium curvature cases compared to flat surfaces. However, a notable decrease in ALDH3A1 expression was observed under high curvature. α-SMA gene expression across all curvature conditions showed a tenfold increase, aligning with the trends noted in immunostaining results. For corneal fibroblasts, ALDH3A1 expression was lower for all curvature cases than that of flat surface, even though it was proportional to the level of curvature. For α-SMA expression, there was a mild increase with curvature increase compared to the flat surface. ALDH3A1 expression in corneal myofibroblasts was lower than that of the flat surfaces, as anticipated. α-SMA expression significantly increased in response to curvature, showing a proportional relationship. These findings suggest that curvature plays a crucial role in modulating cell phenotypes, particularly α-SMA, which is responsive to curvature induced stresses, triggering various upstream or downstream pathways.

### 2.4. Focal adhesion distribution and mechanobiological marker expression

**Figure 4.**
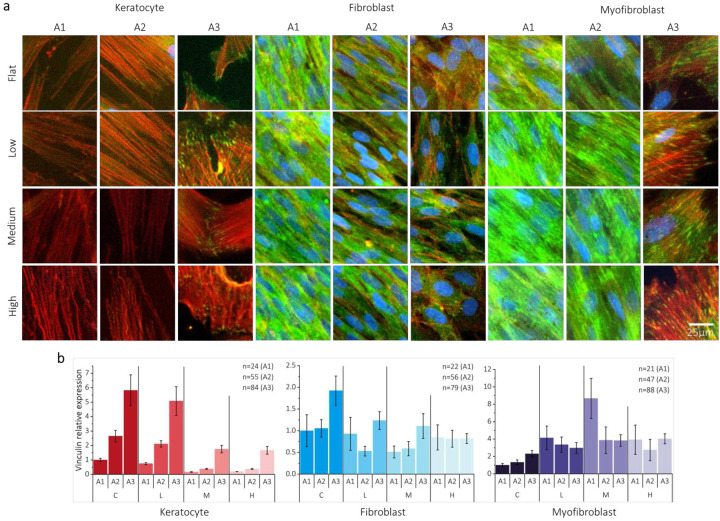
The curvature-influenced focal adhesion. (a) Vinculin stained 40 × magnification images of all cell types on D9 showed a significant difference between slope (A2) and edge (A3) location. The expression on edge (A3) location for all cell types showed the increase of vinculin compared to center (Green: Vinculin, Red: F-actin, Blue: Nuclei). (b) The quantitative expression of vinculin showed on three locations (A1: center, A2: slope, A3: edge). This trend was distinct in cornea keratocyte but myofibroblast showed the opposite trend on curvature.

Vinculin, a key component of focal adhesions, plays a critical role in transmitting mechanical forces, such as generating traction and mechanical forces between cells and ECM^24^. Fig. 4 **and Supplementary Fig. 6** shows the expression pattern of vinculin within actin fibers on D6 and D9, which varied depending on the specific cell type and curvature. As shown in **Supplementary Fig. 6a**, corneal keratocytes showed relatively lower vinculin expression than corneal fibroblasts and myofibroblasts on a flat surface. In medium- and high-curvature cases, vinculin expression in corneal keratocytes was found to be decreased compared to cells cultured on low-curvature and flat surfaces, as shown in Fig. 4a. The decrease in vinculin expression can be considered a potential consequence of phenotypic changes characterized by reduced cell adhesion^25, 26^. Corneal fibroblasts also showed a similar trend to corneal keratocytes, with increased vinculin expression with curvature on D6 but a decrease in vinculin expression with curvature on D9 in **Supplementary Fig. 6b**. In contrast, corneal myofibroblasts showed a significant increase in vinculin expression in all curvatures on both D6 and D9 (**Supplementary Fig. 6b**). This result demonstrates the contractile nature of corneal myofibroblasts under curved substrates. Notably, vinculin expression on D6 was higher overall, which differed from the trend observed on D9 in corneal keratocytes and fibroblasts (**Supplementary Fig. 6b**). The expression of α-SMA in Fig. 3c demonstrates this trend. α-SMA expression of all cell types on D6 clearly exhibited an increasing trend due to curvature, although the values were significantly lower than those on D9.

Vinculin expression in corneal keratocytes was influenced by its location on the corneal curvature. **Supplementary Figures 8 and 9** show the difference in stress between the center (A1), slope (A2), and edge (A3) regions with the percentage of meridional stress to hoop stress of the curvatures on the three locations identified in **Supplementary section 3**. For all three different curvature cases, the hoop stress increased in the center (A1) area in **Supplementary Fig. 9a**. In contrast, the edge (A3) area exhibited the stronger meridional stress rather than hoop stress in **Supplementary Fig. 9b**. **Supplementary Figure 9c** shows the sensitivity of hoop and meridional stress with respect to arc length from peak to trough. At the peak (A1), the percentage of meridional stress over the hoop stress exhibited an inner 5 % on all curvatures. In contrast, at the slope (A2) region, the percentage of meridional stress over the hoop stress shows levels between 5 to 40 %. The edge (A3) area showed the highest percent of meridional over hoop stress by showing levels between 40 to 150 %. This trend was increasingly distinct on the increase of curvature. The increasingly enhanced meridional stress close to the edge area and considerable fluctuation in the edge region affected the vinculin expression depending on the location of curvature. When comparing corneal keratocytes located on the slope and edge regions, significant differences were observed in the distribution of focal adhesions and cell morphology, as depicted in Fig. 4. The activated corneal fibroblasts, except for high curvature, showed a similar vinculin expression pattern as corneal keratocytes, with increased vinculin expression on the edge (A3) starting from the center (A1), as depicted in Fig. 4b. However, in the high-curvature case, this trend was reversed in corneal fibroblasts. In contrast to corneal keratocytes, corneal fibroblasts demonstrated comparable levels of vinculin expression at all three high curvature locations. However, myofibroblasts displayed distinct opposing trends in the low and medium curvatures, with the highest level of vinculin expression in the A1 area. Myofibroblasts with high curvature showed a statistically similar trend in all areas, similar to the trend observed in corneal fibroblasts with high curvature. This implies that the sudden increase showing the highest meridional stress over hoop stress in the edge (A3) area on high curvature affects corneal fibroblasts and myofibroblasts by modulating the focal adhesion trend. This trend may be related to the high contractility of myofibroblasts, leading to increased focal adhesions, which is directly influenced by curvature. Moreover, enhanced A3 compared to A1 and A2 on even the flat surface of all cell types, rather than medium and high curvatures, implies that collective migration can also affect focal adhesion.

In **Supplementary Fig. 10**, cornea keratocytes showed a distinct decrease of expression of Rho A in response to curvature and the constant expression of actin-related protein 2/3 complex subunit 2 (ARPC2). Corneal fibroblasts exhibited constant expression of both ARPC2 and RhoA, whereas corneal myofibroblasts exhibited a decreasing trend in ARPC2 expression and enhanced RhoA. As shown in Fig. 4 and **Supplementary Fig. 6**, corneal keratocytes and fibroblasts on the slope exhibited organized vinculin expression at the head and tail of the elongated cells, in contrast to the diffuse and unorganized vinculin expression observed in myofibroblasts. These findings are supported by ARPC2 expression, which is related to cell elongation and lamellipodia. Conversely, RhoA expression showed an opposite trend to that of ARPC2. Unlike corneal myofibroblasts, corneal keratocytes act downstream of RhoA expression. Corneal myofibroblasts showed higher Rho A expression at high curvature compared to low, medium, and flat of myofibroblasts. On flat cornea keratocytes shown in Fig. 4b, vinculin expression at the edge (A3) indicated that collective migration was the reason of the RhoA expression and linked to increased vinculin expression at the A3 area. However, these expressions were significantly decreased by curvature. In contrast, corneal myofibroblasts enhance the formation of stress fibers and focal adhesions while expressing higher levels of Rho A by curvature.

### 2.5. Effect of curvature on cell alignment

**Figure 5.**
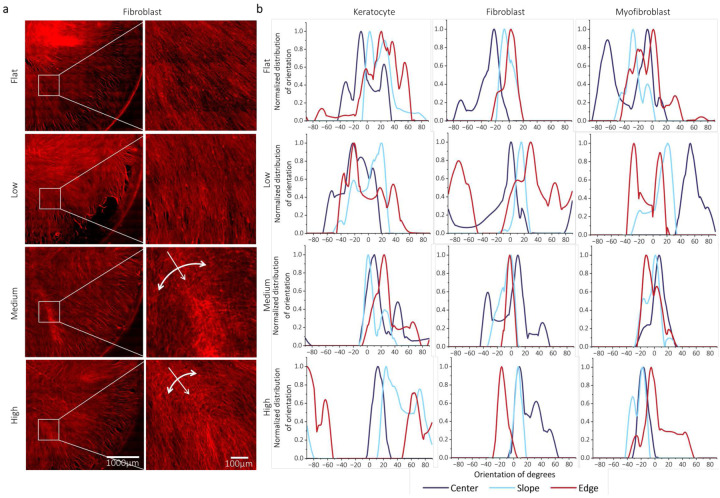
Influence of curvature on the cell alignment of cornea stromal cells. (a) F-actin tile images of fibroblasts showing an organized orthogonal cell pattern on medium and high curvature compared to flat. (b) The normalized distribution of the cell orientation on center, slope, and edge of each curvature.

Figure 5 depicts the cell alignment in the center, slope, and edge areas, and their orientations, whereas **Supplementary Fig. 11** presents the overall orientation of all stromal cell types across the three distinct curvatures. In Fig. 5a, the F-actin of corneal fibroblasts shows variations in cell orientation in response to the level of curvature and cell orientation at three different locations (center, slope, and edge) for all cell types. The overall distribution of all cell types on flat and low-curvature surfaces showed a trend with random and multiple peaks at three locations, indicating a lack of consistent cell orientation. In contrast, all cells with medium and high curvatures showed preferential alignment and polarization in the direction of the curvature. As shown in Fig. 5b, the medium and high curvatures exhibited a more focused distribution with a single peak at three locations, suggesting a more aligned cell orientation in response to these curvatures. In **Supplementary Fig. 9c**, the percentage of meridional stress over hoop stress significantly increased on the slope (A2) area than the center (A1) area. This indicated that the cell pulling stress toward the longitudinal direction was more enhanced at the A2 area. The highest percentage of meridional stress over hoop stress was observed in the edge area (A3), including the random stress arrows in **Supplementary Fig. 9a and b**. The random stress and the strongest stress at the edge of the curvature were enhanced by increasing the curvature. This increased random stress on the high curvature, unlike other curvatures, can affect the phase shifting of cell orientation on specific edge areas of corneal keratocytes due to sensitivity^27^. This is supported by the finding that corneal keratocytes expressed lower vinculin expression on medium and high curvatures, although increased α-SMA expression was observed on medium and high curvatures.

In the medium- and high-curvature cases, we observed orthogonal cell alignment of corneal fibroblasts, which is a unique pattern of native ECM alignment on the basal side of the corneal stroma. As indicated in Fig. 5a, the medium-curvature case showed 99.99° actin fiber crossing, and the high-curvature case showed 88.65° crossing. In addition, the orthogonal pattern showed location dependency. The orthogonal crossing of the actin fibers did not appear at the top and center, but the slope showed visible orthogonal crossing. This trend was consistent with the simulation of meridional and hoop stress shown in **Supplementary Fig. 9c.** The alignment of cornea fibroblast cells exhibited a distinct pattern depending on the percentage of dominant stress. On the center (A1) area, the hoop stress, which was significantly dominant, induced the random cell orientation in **Supplementary Fig. 9a and c**. On the other hand, the meridional stress gradually increased starting from the point inner around 1mm diameter to the edge in **Supplementary Fig. 9c**. The outer around 3.5mm diameter line showed an abrupt decrease in meridional stress in **Supplementary Fig. 9c**. These critical points showed the correspondence with the baseline established for determining the location of center, slope, and edge, as detailed in **Supplementary Section 3**. The transverse stress hindering the pulling stress toward the bottom was governed by the percentage of meridional stress to hoop stress, shown in **Supplementary Fig. 9c**. The overall transverse stress was strongest in the center area, decreasing smoothly towards the slope and edge areas as the curvature increased.

**Supplementary Figure. 11a** shows the orientation of each stromal cell captured within a specific region of the tile images (toward 45° direction of quarter sized circle). The orientation of the cells was influenced by three different curvatures, and the overall organization of the cells was observed to be influenced by the curvature compared to the flat surface. As depicted in **Supplementary Fig. 11b**, the cell orientation on the flat and low curvatures displayed broad and unorganized peaks for all cell types, whereas the other curvatures showed shallow and organized peaks. This suggests that curvature can significantly affect cell orientation, regardless of the cell type. These findings highlight the role of curvature in guiding cell alignment and emphasize the universal influence of curvature on cell behavior and organization irrespective of the specific cell type.

### 2.6. Effect of curvature on ECM expression

**Figure 6.**
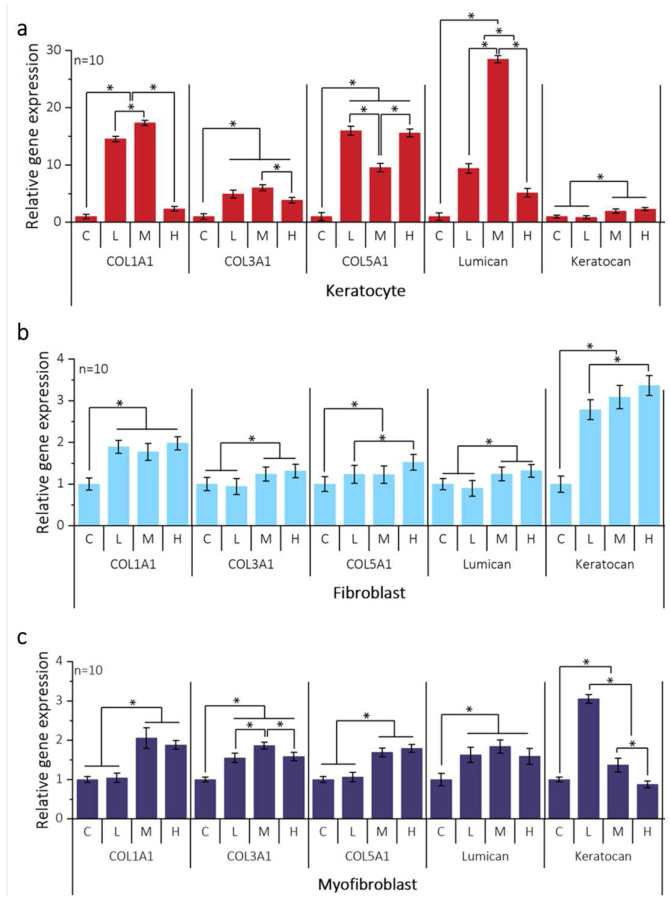
Effect of curvature on gene expression of extracellular matrix in cornea stromal cells. Using real-time quantitative reverse transcription polymerase chain reaction, the relative transcript levels of each cell type on D9 were normalized with GAPDH and quantified using the 2^−ΔΔCt^ method compared to the flat. Markers associated with extracellular matrix deposition, including lumican, keratocan, collagen I, collagen III, and collagen V, were either upregulated or downregulated in response to curvature. GAPDH, glyceraldehyde 3-phosphate dehydrogenase.

The ECM in the corneal stroma, which consists mainly of collagen and proteoglycans, provides the mechanical integrity and arrangement of the cornea stroma^28^. As shown in the collagen type I immunostaining images in **Supplementary Fig. 12**, unlike corneal myofibroblasts, corneal keratocytes do not express collagen type I in response to curvature. **Supplementary Figure. 12a** shows the expression of type I collagen with a distinct trend among the different cell types. Corneal keratocytes and fibroblasts showed relatively low expression levels of type I collagen compared to corneal myofibroblasts. In **Supplementary Fig. 12b**, the quantitative analysis of collagen type I expression in response to the curvature was performed on D6 and D9. None of the cell types showed an increase in collagen type I expression until D6. On D9, there were no significant differences in type I collagen expression between corneal keratocytes and fibroblasts across the various curvatures. However, corneal myofibroblasts exhibited notably higher collagen expression than flat myofibroblasts on D9. Interestingly, these myofibroblasts presented elevated collagen type I expression levels, which aligned with the trend observed for vinculin expression, as shown in **Supplementary Fig. 6b**. Furthermore, the gene expression of collagen types I, III, and V was assessed to understand how ECM deposition is induced by curvature. As shown in Fig. 6a, the corneal keratocytes showed an overall increase in collagen expression starting from the flat to medium curvature. However, for high curvature cases, there are a noticeable decrease in collagen type I expression. This suggests that curvature levels exceeding the norm could result in the reduction of collagen deposition. A similar trend was found in collagen type III in corneal keratocytes, unlike corneal fibroblasts and myofibroblasts. In Fig. 6b and c, corneal fibroblasts and myofibroblasts showed significantly increased collagen type I, III, and V expression on medium and high curvatures, while collagen type III of corneal fibroblasts on low curvature and collagen types I and V of corneal myofibroblasts on low curvature showed consistent expression levels with the flat.

Additionally, quantitative gene expression analysis was performed for two SLRPs (lumican and keratocan). Fig. 6a shows that lumican expression in corneal keratocytes increased progressively with curvature, reaching its highest level at a medium curvature. However, high curvature resulted in lower lumican expression than medium curvature, a pattern similar to that of collagen types I and III. In contrast, keratocan expression in corneal keratocytes was slightly higher at the medium and high curvatures than at the low and flat curvatures, as shown in Fig. 6a. In the corneal fibroblasts of Fig. 6b, the relative expression level of lumican by curvature compared to flat was lower than that in the corneal keratocytes. However, corneal fibroblasts exhibited slightly higher levels of lumican expression at medium and high curvatures, similar to keratocan expression in corneal keratocytes. In addition, the expression of keratocan in corneal fibroblasts showed a greater difference in curvature, with increasing expression levels of keratocan in response to increasing curvature (Fig. 6b). However, unlike corneal keratocytes and fibroblasts, corneal myofibroblasts exhibit different expression trends of lumican and keratocan on different curvatures. The expression of lumican in corneal myofibroblasts on curvature was significantly higher than that on the flat, as shown in Fig. 6c. Keratocan expression in corneal myofibroblasts showed the highest level of low curvature but significantly decreased starting from the medium curvature (Fig. 6). Taken together, the curvature distinctly affects all stromal cells during ECM development. Corneal keratocytes actively reacted to keratocan expression by matching the increase in curvature; however, lumican expression was downregulated at high curvature. Corneal fibroblasts showed significantly increased keratocan expression, while lumican expression slightly increased starting from the medium curvature. In contrast, corneal myofibroblasts have a critical response to curvature, characterized by increased expression of both lumican and keratocan; however, keratocan expression decreases significantly from medium curvature onward.

## 3. DISCUSSION

Our findings highlight the critical role of curvature on cell proliferation, cell phenotype, organization, and matrix reconstruction. **The proliferation profiles** clearly show that curvature significantly dictates cell growth and their distribution over surfaces. From the mechanobiological view, this indicates that increased curvature leads to higher tensional forces, which promotes cell migration. Previous experiments with PA-coated surfaces have shown that stromal cells migrate from the outer edges to the apex of the curvature, creating a highly aligned cell pattern^29^. Without PA coating, the cells exhibit an inability to migrate energetically unfavorable gradient of the curved substrate. Contrarily, stromal cells cultured on the apex of the curvature were able to proliferate and migrate with significant alignment. This result implies that stromal cells have an intrinsic ability of stromal cells to perceive curvature gradients, adjusting their proliferation and migration towards energetically optimal trajectories^30^.

### For the phenotype investigation

For the phenotype investigation, we observed that curvature alone, without any chemical treatments, induced cell phenotype transformation. Typically, cornea keratocytes are known non-contractile cells, characterized by high ALDH3A1 and low α-SMA expressions. However, in response to curvature, we observed phenotypic changes in corneal keratocytes, notably an increase in α-SMA expression. This suggests that curvature alone can trigger phenotypic alteration in corneal keratocytes towards a myofibroblast-like phenotype without any chemical treatments. Typical α-SMA signaling pathways are closely associated with the activation of the TGF-β/Smad2/3 signaling pathway^31, 32^. Our findings show that mechanical stimuli can stimulate TGF-β/Smad2/3 signaling pathways, which can be explained by exploring crosstalk between Rho/ROCK and Smad2/3 pathways^33 34, 35^. Even though recent studies have shown the possible crosstalk between mechanical strain influences the Rho/ROCK pathway^33^ and the Smad2/3 signaling pathway^34, 35^ in lung, kidney, and sclera^33–38^, the precise mechanisms by which ROCK influences the expression of TGF-β-induced profibrotic mediators are yet to be uncovered. To accurately determine the impact of these phenotypic changes in corneal keratocytes, further in-depth analyses at the protein and gene levels are required to dissect the signaling pathways involved. In contrast to α-SMA, the expression of ALDH3A1 in quiescent form cornea keratocytes notably reduced, deactivating the pathway to trigger ALDH3A1 under high curvature. ALDH3A1 is a known crystalline protein that maintains the transparency of the cornea stroma^36^. This downregulation of ALDH3A1 in cornea keratocytes under high curvature conditions may be associated with the stromal cloudiness observed in keratoconus. Additionally, cornea fibroblasts and myofibroblasts exhibit a similar response to curvature, characterized by increased expression of α-SMA. With these overall expression trends, we conclude that the curvature has an antagonistic effect on ALDH3A1 expression and agonistic effect on α-SMA expression in cornea stroma cells.

### The contractile properties

The contractile properties of corneal stroma cells are closely linked to the expressions of α-SMA^37^ and the development of focal adhesions^38, 39, 40^. Interestingly, the focal adhesion in quiescent cornea keratocytes and activated fibroblasts exhibited the opposite trend with myofibroblast when expressing a considerable rise in α-SMA on curvature for late phase (D9). This indicated that a slight increase in α-SMA expression for early phase (D6) may correspond to increased vinculin expression in cornea keratocytes and fibroblasts. Conversely, the considerable increase α-SMA expression for late phase (D9) seems to coincide with the initiation of phenotypic changes in corneal keratocytes and fibroblasts accompanied by reduced vinculin expression. Previous studies have found that phenotypic change downregulated the vinculin expression due to cell motility^41^. This suggests a potential relationship between α-SMA expression and the downregulation of vinculin for late phase in the case of cornea keratocytes and fibroblasts due to phenotypic change. Contrary to cornea keratocyte and fibroblast, corneal myofibroblasts are highly responsive to curvature-induced mechanical stress, as indicated by the upregulation of α-SMA and vinculin expression. Focal adhesions are often associated with activities of Rho GTPase family proteins and actin-related protein 2/3 complex (Arp2/3), which act as mechanosensors for regulating cell migration, cell morphology, and adhesion^42^. During migration, cells exhibit stronger RhoA activity-associated filopodia and traction forces, which pull follower cells. Conversely, in follower cells, Arp2/3 expression is linked to the formation of lamellipodia, sensing neighboring cells^43^. Observing the curvature effect on cell migration through ARPC2 and RhoA, corneal keratocytes exhibit a decrease in RhoA expression compared to those on flat surfaces, a trend similar to that of vinculin expression. Corneal fibroblasts maintain a similar ratio of ARPC2 and Rho A, while the medium curvature enhances the expression levels of both compared to other curvature cases. In contrast, corneal myofibroblasts display the opposite trend, suggesting a correlation between vinculin expression and RhoA, and indicating that myofibroblast elongation, associated with ARPC2, is not significantly affected by curvature. This finding suggests that each stromal cell type has a distinct preference for its role in interactions with adjacent cells and substrates. Corneal keratocytes prioritize cell-to-cell communication, showing sensitivity to the surrounding environment, such as movements of adjacent cells and variations in cell density, rather than generating traction force for cell migration. Corneal fibroblasts balance interactions between cell-to-cell communication and the generation of pulling forces by interacting with substrates. Corneal myofibroblasts, however, favor cell-to-substrate communication, exerting stronger stress instead of focusing on cell-to-cell communication. A detailed follow-up study could reveal the specific behavioral differences due to curvature, particularly the role of Cdc42, the upstream regulator of the Arp2/3 signaling pathways, in controlling cell-cell communication during cell migration^44, 45^.

### Cell alignment

Cell alignment in response to curvature is closely associated with the mechanical properties of the curvature. On curved substrates, the slope presents a high net displacement, which induces cell traction forces^46^, while the edge of the curvature experiences high stress^47^. Furthermore, curved substrates typically have two stress components: meridional and hoop stresses^48, 49^. For all stromal cells, cell alignment is significantly affected by medium and high levels of curvature, compared to the minimal impact seen with low curvature and flat surfaces. Among the stroma cells, cornea fibroblasts show more sensitive to the curvature than other types of cells. The cell orientation of cornea fibroblasts on low curvature was influenced by dominant hoop stress, resulting in less organized cell alignment and random patterns. However, medium and high curvature cases, the hoop stress is dominant over meridional stress while the pulling force is enhanced. Thus, cells were aligned the direction of hoop stress, suggesting that the harmony of meridional and hoop stress critically affects the cell alignment. Notably, cell alignment of cornea fibroblasts projected the meridional and hoop stress induced by curvature, showing an orthogonal pattern, unlike other cells. The significance of this finding is noteworthy when comparing it to that of previous studies that have explored the orthogonal pattern of cornea stromal cells using ascorbic acid and special patterned substrate^50, 51^. Without ascorbic acid or any surface patterns, we observed an orthogonal pattern in corneal fibroblasts, implying that the unique mechanical environment created by the curvature significantly contributes to the organization of this orthogonal pattern.

### The curvature-induced expression of ECM

The curvature-induced expression of ECM in stromal cells is consistent with their role in ECM deposition and remodeling. Within the corneal stroma, collagen types I and III are fundamental to its structure and elasticity, while collagen type V plays a critical role in regulating the formation and assembly of type I fibrils^52, 53^. Curvature was found to have a significant impact on the expression of collagens in all corneal stroma cells. For corneal fibroblast and myofibroblast, the gene expressions of collagen I, III, and V increase with an increase in curvature. It is well known that biomechanical strain often leads to increased collagen synthesis by fibroblasts, as part of the natural healing and remodeling process, where the extracellular matrix (ECM) is reinforced to withstand mechanical demands. Similar trend was observed with curvature which is closely linked with corneal stroma remodeling processes^54^. However, only corneal keratocytes on high curvature showed decreased expression in collagen types I and III, even though the collagen type V expression increased. This trend suggests that high curvature may induce corneal keratocytes to suppress the formation of the primary collagen matrix and engage more regulatory pathways of collagen synthesis, likely aiming at preventing excessive collagen accumulation. Furthermore, the expression levels of lumican and keratocan are significantly influenced by the degree of curvature, with high curvature conditions resulting in the downregulation of both lumican and keratocan. These downregulations lead to significant alterations in the structural integrity of the cornea. Previous knockout mice studies found that lumican deficiency causes cloudiness or opacity in the corneas. This cloudiness is attributed to changes in the collagenous matrix, characterized by larger fibril diameters and disorganized fibril spacing^55, 56^. Furthermore, Keratocan knockout mice exhibit relatively thin and transparent corneas, and minimal alterations are observed in the stromal collagenous matrix^57, 58^. This suggests that the loss of lumican and keratocan under high curvature has potential links to cornea diseases associated with shape deformation, leading to cloudiness and a disorganized structural arrangement.

Curvature plays a critical role in the homeostasis of stromal cells which helps to maintain a healthy cornea stroma and remodeling in progressing cornea shape deformation. In this study, we show that curvature impacts on cell proliferation, phenotype, alignment, migration, as well as synthesis and deposition of ECM. Various corneal pathologies are connected to curvature. A key example is keratoconus, a non-inflammatory condition where the cornea thins and gradually bulges into a cone shape. This bulging can lead to vision distortion and, in severe cases, blindness^59, 60, 61, 62^. The keratoconus progression causes particularly the loss of keratocytes, leaving the stroma more vulnerable to external stress. For keratoconus, the increased expression of collagen and α-SMA due to abnormal curvature is identified as a crucial marker^63^. This excessive expression of collagen and proteoglycans by curvature impacts the dehydration resistance and mechanical strength of the corneal stroma, thus contributing to the progression of keratoconus^64^. The condition is marked by heightened α-SMA expression, enhanced focal adhesion, and ECM deposition triggered by curvature, all of which significantly correlate with the progression of keratoconus^65, 66^. However, further research is needed to elucidate the effects of curvature at the molecular level, particularly the long-term impact of curvature on corneal dysfunctions and wound healing processes. This includes exploring the molecular mechanisms related to ECM deposition and degradation, influenced by corneal shape deformation, by examining the activities of tissue inhibitors of metalloproteinase (TIMP) and matrix metalloproteinase (MMP)^67^. Such studies promise to yield valuable insights, enriching our understanding of the mechanisms behind corneal stroma shape dystrophy.

## METHODS

### 4.1. Microfabrication of a hydraulically controlled curvature chip array

The hydraulically controlled chip array was designed and fabricated to generate diverse cornea-like curvatures on planar surfaces. The chip comprises three main components: a hydraulic layer, thin polydimethylsiloxane (PDMS) membrane, and medium reservoir. Utilizing the cornea anatomy data^68^, the design of the 4×5 hydraulic array layer was created using AutoCAD (**Supplementary Fig. S13**), and the pattern was then cut on a 250 μm silicone polymer PDMS sheet (BISCO HT-6240, Rogers Corp, USA) using a Roland cutter (GX-24, Roland DGA Corp, USA). For both the thin PDMS membrane and medium reservoir, PDMS (SYLGARD 184, DOW Corning, USA) was prepared by mixing the base and cross-linker agent at a ratio of 10:1. The mixture was degassed in a vacuum chamber for 30 min. Subsequently, the PDMS mixture was poured onto a 150 mm petri dish and cured overnight at a temperature of 60 °C in a dry oven, resulting in a 5-mm thick PDMS layer. The medium reservoir was formed by punching a 10-mm diameter hole into the cured PDMS layer. A 10:1 PDMS solution was spin-coated at 1,100 rpm for 15 s to create a thin PDMS membrane. This process resulted in a 100 μm PDMS layer. The curing procedure used for the thick PDMS layer was employed to prepare a thin PDMS membrane. Each chip layer was bonded sequentially after the oxygen plasma treatment using a plasma etcher (PE-25, Plasma Etch Inc.). Initially, a microfluidic pattern for hydraulic actuation was bonded to a 50 × 75 mm glass slide. Subsequently, a thin PDMS membrane was placed on top of the hydraulic layer after oxygen plasma treatment. Finally, the medium reservoir was bonded to the top of the PDMS membrane to form the culture reservoir. The assembled chip was placed on a hot plate at 80 °C for 12 h to enhance the bond strength. Before cell culture, all chips were sterilized with 80% ethanol and rinsed with DIW.

### 4.2 Control and characterization of the curvature array chip

The thin PDMS layer formed three different curvatures (low, medium, and high), which allowed it to be inflated by injecting DIW through the inlet port of the hydraulic layer using a syringe (309659, BD, USA). The detailed procedure is described in **Supplementary Section 4**. The thick PDMS reservoir layer provided adequate structural support to prevent delamination owing to the high pressure from the hydraulic chamber, thereby sustaining the desired curvature. By capturing the angle profiles during hydraulic injection, the relationship between the curvature and injection volume was defined using a stereo microscope equipped with a Ximea CCD camera (model MQ042RG-CM, Ximea), as shown in **Supplementary Fig. 1**. The hydraulic chamber was initially filled with 45 μL of water, and then water was continuously added at a rate of 60 μL/min. The captured images were analyzed using ImageJ software to determine the angle of curvature. To measure the angle of curvature, a reference baseline consisting of a horizontal line and 90° vertical line was established. All images were captured from the same position, with the same baseline as that of the reference. Three specific points were marked using the region of interest (ROI) manager in ImageJ: 1) the top position of the dorm-shaped curvature; 2) the point where the curvature intersects the horizontal line; and 3) the midpoint of the intersection line was connected to the second line. By marking these three points, angle (A) in the image was measured using ImageJ software. The measured angle was calculated using circular segment formulas and converted into diopters. Detailed characterization of the three curvatures is presented in **Supplementary Section 5**.

### 4.3. Surface functionalization for cell culture

Surface modification of PDMS was necessary for stable cell culture under curvature because of its high hydrophobicity. Polydopamine (PDA) was applied to PDMS to enhance the ECM bond strength. Dopamine (DA) acts as a covalent linker, bonding collagen to PDMS by forming a PDA layer through oxidative polymerization with the polymer chains of PDMS. With the PDA-assisted ECM coating presented in the detailed condition of **Supplementary Section 1**, cells stably formed a layer under a high-strain-curved PDMS substrate. First, the PDMS surface was treated with oxygen plasma for 1 min using a plasma etcher (PE-25, Plasma Etch Inc.). Subsequently, a solution containing dopamine hydrochloride (0.01% w/v, H8502, Sigma-Aldrich, USA) in a 10 mM Tris-HCl with a pH 8.5 (MB-027–1000, ROCKLAND, USA) was applied to the surface, followed by a 24 h immersion. After this coating, the surfaces were washed twice with DIW (high quality, 18.5 MΩ) to eliminate any unbound dopamine molecules. The treated surfaces were then allowed to air dry. Following this, 100 μg/mL collagen type I (5005, PureCol^®^, Advance biomatrix, USA) solution was prepared and added over the dopamine-coated surface overnight. The PD-coated PDMS was rinsed twice with DIW and UV-sterilized for 1 h before the cell culture.

### 4.4. Cell culture and droplet cell seeding

Primary human corneal keratocytes (HCK, 6520, ScienCell, USA) were cultured in Fibroblast Medium (2301, ScienCell, USA) containing 2% fetal bovine serum (FBS; #0010, ScienCell, USA), 1% FCS (#2352, fibroblast growth supplement, ScienCell, USA), and 1% P/S (#0503, antibiotic solution, ScienCell, USA) on a poly-L-lysine (10 mg/ml, #0413, ScienCell, USA) coated flask by the third passage. Cultured keratocytes were diluted with the medium to prepare approximately 2.5 × 10^5^ cells/mL and 1 × 10^6^ cells/mL. Each cell concentration was loaded into 3 mL empty cartridges (CSC010300102, CELLINK Inc.), and droplet cell seeding was performed at the center of each well of an 8 mm curvature chip by controlling the 3D bioprinter (Bio X^™^ CELLINK Inc.) with a 25 G needle mounted injector. The initial cell coverage on the chip surface was analyzed after staining cells with Hoechst 33258 (H3569, Invitrogen, USA) for two different densities (2.5 × 10^5^ cells/mL and 1 × 10^6^ cells/mL), and the cell coverage density (cells/cm^2^) was quantified using ImageJ by counting the Hoechst-stained cells and measuring the cell coverage area. This analysis involved counting the initial number of cells and measuring the coverage area within the image. The detailed measured procedure of analysis is presented in **Supplementary Section 6, 7 and 8**.

### 4.5. Cell transformation: Corneal fibroblasts and myofibroblasts

Using different media, quiescent corneal keratocytes were differentiated into activated corneal fibroblasts and myofibroblasts on a curved array chip. The quiescent corneal keratocytes were cultured in serum-starved medium containing DMEM/F12 (11320033, Gibco, USA) supplemented with 1% penicillin/streptomycin (15140122, Gibco, USA), 1 mM L-ascorbic acid 2-phosphate (49752, Sigma-Aldrich, USA), and 1% insulin transferrin selenium solution (I3146, Sigma-Aldrich, USA). To transform the keratocytes into activated corneal fibroblasts, DMEM/F12 supplemented with 10% FBS, 1% penicillin/streptomycin, and 10 ng/mL recombinant human basic fibroblast growth factor (bFGF) (233-FB, R&D Systems, USA) was used to maintain the fibroblastic phenotype. Finally, to obtain cornea myofibroblasts, the fibroblasts were differentiated using a medium containing DMEM/F12, 10% FBS, and 10 ng/mL recombinant human transforming growth factor (TGF-β) (240-B-002, R&D Systems, USA). Three different cell types were cultured for three days on a planar surface, and the phenotype of each cell was confirmed before applying curvature.

### 4.6. Immunostaining and image acquisition

Each well of the array chip was washed with phosphate buffered saline (PBS) thoroughly and fixed in 10% formalin for 15 min. After washing three times with PBS, all curved chips were deflated to form a flat shape after the fixation procedure for high-quality fluorescent imaging. Fixed cell layers were then permeabilized in 0.1% Triton X-100 for 15 min at room temperature. To immunostaining intracellular proteins such as α-SMA and ALDH3A1, a 1:1 solution of acetone and methanol for fixation and permeabilization at −20 °C was performed to enhance visualization of these proteins instead of using formalin and Triton X-100. After these treatments, the samples were washed with PBS, blocked with 1% bovine serum albumin (BSA) at room temperature for 1 h, and then incubated with primary antibodies overnight at 4 °C. The samples were washed thrice with PBS, incubated with secondary antibodies for 1 h at room temperature, and washed thrice with PBS to remove unbound antibodies. Finally, the cells were stained with Hoechst 33258 (H3569; Invitrogen). The primary and secondary antibodies are listed below in **Supplementary Table 2**: Primary-TRITC Rhodamine phalloidin (P1951, Sigma-Aldrich, diluted 1:100), mouse anti-collagen type 1 (MAB3391, diluted 1:100), rabbit anti-vinculin (ab129002, Abcam, diluted 1:100), rabbit anti-ALDH3A1 (ab76976, Abcam, diluted 1:100), mouse anti-α-SMA (ab7817, Abcam, diluted 1:100), and secondary-goat anti-mouse Alexa Fluor 488 (SAB4600388, Sigma-Aldrich, diluted 1:200) and goat anti-rabbit Alexa Fluor 594 (A-11012, Invitrogen, diluted 1:100). Cell imaging was performed using a Nikon Eclipse Ti fluorescence microscope with the NIS Element software. The cell layers in each well of the array chip were imaged at 40 × magnification and merged into one tile image. All images were captured with the same exposure time and fluorescence settings to compare the expression levels of each group.

### 4.7. Immunofluorescence image analysis

A quarter-sized image (8 mm diameter circle) made from a collection of 40 × magnification images was divided into smaller, equally sized images (1024 × 1024 pixels) for analysis using MATLAB (R2022b). Custom MATLAB code was developed to quantify and compare the mean green and red fluorescence intensities for immunostaining of multiple individual images. The mean intensity and the standard deviation of red and green intensities from the number of cells per image were analyzed to compare the expression of ALDH3A1, α-SMA, vinculin, and collagen type I affected by the curvature. All images were preprocessed by Gaussian filtering and image thresholding and analyzed after the RGB split. Both the red and green channels of the cell images were subjected to background subtraction using a rolling-ball algorithm with a radius of 10–20 pixels. Fluorescence was quantified using Equation (1) below^69^:

Correlatedtotalcellfluorescence(CTCF):CTCF=[integrateddensity]–[areaofdesignatedcell'smeanbackgroundfluorescencereadings]

The mean CTCF fluorescence intensity of all individual images was calculated by dividing by the total number of nuclei. Overall quantification was performed by averaging the values of all images within the two tile images. Furthermore, localized quantitative immunofluorescence was analyzed based on the two boundaries of the net displacement profiles, as explained in **Supplementary Section 3**.

### 4.8. Cell alignment analysis

By selecting a localized 1280 × 1420 μm area at the center, slope, and edge of the curved surface from a quarter-sized tile image, cell alignment of the curvature array chip was analyzed with 40 × f-actin and DAPI images. Cell orientation was analyzed using the OrientationJ (Biomedical Imaging Group, EPFL, Switzerland) plug-in in ImageJ (NIH, Bethesda, Maryland, USA) software^70^. The gradient option of the cubic spline with a specified neighborhood was employed to estimate the discrete gradient at a given point in OrientationJ. By adjusting the threshold, brightness, and contrast of the images, they were processed into binary containing a dark background and exported as 32-bit TIFF files through ImageJ. The images were analyzed by quantifying the percentage of white pixels representing cells orientation at each orientation angle between −90° and 90° from OrientationJ. The local window in OrientationJ was set to 50 pixels because the size of each cell was equivalent to approximately 50 pixels. The frequency distribution of the cell directions was normalized by calculating the percentage of occurrence for each angle. This normalization was achieved by dividing the frequency of each angle by the highest observed frequency, resulting in a representation in which the maximum frequency was scaled to one. This normalization allows for a standardized comparison of the alignment across different curvature conditions.

### 4.9. RNA isolation and RT-PCR gene level quantification

Ten samples were collected from each cell group, and gene expression was investigated after six days of curvature application. According to the manufacturer’s instructions, RNA was isolated from the harvested cells using the Direct-zol RNA miniprep kit (R2051, Zymo Research, USA). Complementary DNA (cDNA) was synthesized using the High-Capacity cDNA Reverse Transcription Kit (4368814, Applied Biosystems, USA) with 0.2 μg of total RNA. Real-time quantitative PCR (RT-qPCR) was performed using the Power SYBR Green Master Mix (Applied Biosystems, 4367659) using the listed primers in **Supplementary Table 1** to analyze gene expression associated with ECM related markers (COL1A1, COL3A1, COL5A1, Lumican, and Keratocan) and phenotype related markers (ALDH3A1 and α-SMA) along with GAPDH as a reference. Results were relatively quantified as an average fold change compared to flat using the 2^−*ΔΔCt*^ method from a minimum of three biological replicates.

### 4.10. Statistical Analysis

Experimental trials were conducted across four distinct curvature levels (high, medium, and low) in addition to a curvature-free flat group for comprehensive comparative analysis. All datasets obtained from three independent replicates (N=3) and results are presented as mean values with accompanying error bars indicating standard deviation (S.D.), the number of analyzed samples (n>5) being displayed on the figures. The determination of statistically significant variations between groups was facilitated using one-way analysis of variance (ANOVA), accepting P values of less than 0.05 and a 95% confidence interval as indicators of significance in Figure 2b, Figure 3d, Figure 6 and Supplementary Figure 15c. Graphical data representation and statistical assessments were performed using Excel (Microsoft, USA), Origin Pro 2023b (OriginLab corporation, USA), SPSS 29.0 (IBM Analytics, USA), MATLAB R2023a (The MathWorks Inc, USA).

## Data Availability

The authors declare that all data supporting the outcomes of this research are accessible within the article and its Supplementary Information. The MATLAB code for quantifying fluorescent intensity, relevant to this paper, is provided as supplementary material. Any further inquiries or requests for additional information can be directed to the corresponding authors.
